# The Association Between Vitamin D Levels and Erectile Dysfunction in Men: A Systematic Review

**DOI:** 10.3390/jcm14248630

**Published:** 2025-12-05

**Authors:** Radvilė Matukaitienė, Augustė Pikelytė, Birutė Žilaitienė, Robertas Lažauskas, Rasa Verkauskienė, Jonas Čeponis

**Affiliations:** 1 Institute of Endocrinology, Lithuanian University of Health Sciences, Eivenių Str. 2, LT-50161 Kaunas, Lithuania; radvile.matukaitiene@lsmu.lt (R.M.); birute.zilaitiene@lsmu.lt (B.Ž.); rasa.verkauskiene@lsmu.lt (R.V.); 2 Medical Academy, Lithuanian University of Health Sciences, A. Mickevičiaus Str. 9, LT-44307 Kaunas, Lithuania; auguste.pikelyte@stud.lsmu.lt; 3 Institute of Physiology and Pharmacology, Medical Academy, Lithuanian University of Health Sciences, A. Mickevičiaus Str. 9, LT-44307 Kaunas, Lithuania; robertas.lazauskas@lsmu.lt

**Keywords:** vitamin D, 25-hydroxy-vitamin D, erectile dysfunction, hypogonadism

## Abstract

**Background/Objectives:** Erectile dysfunction (ED) is a common sexual disorder in men, frequently linked to endothelial dysfunction affecting penile vasculature. Accumulating evidence suggests that vitamin D (VD) status may influence endothelial function and, consequently, erectile function. VD deficiency has also been associated with cardiovascular risk factors, which are well-known contributors to ED. **Methods:** A systematic review following PRISMA guidelines was conducted, analyzing studies from PubMed and Cochrane databases published between 2010 and 2025. Randomized controlled trials, observational studies, and pilot clinical trials examining the relationship between VD levels and ED in the general male population were included. **Results:** Out of 1335 identified articles, 10 studies met inclusion criteria, encompassing over 13,000 men. Observational studies consistently showed that men with moderate-to-severe or arteriogenic ED had significantly lower serum VD levels and poorer erectile function scores compared to those with mild ED. VD deficiency was independently associated with higher ED prevalence, irrespective of lifestyle, cardiovascular risk, or sex hormone levels. Although several observational studies suggested a potential optimal vitamin D threshold, definitive recommendations cannot be established due to the heterogeneity of available evidence and conflicting findings from randomized controlled trials. The latter demonstrated inconsistent effects of vitamin D supplementation on erectile dysfunction outcomes, with the largest trial reporting no significant reduction in disease prevalence. These findings underscore the critical need for rigorously designed trials targeting populations with severe VD deficiency and arteriogenic ED. **Conclusions:** This systematic review highlights an association between vitamin D status and erectile dysfunction, particularly in men with moderate-to-severe or arteriogenic ED. However, most of the evidence is derived from low-certainty observational studies. While observational data suggest potential benefits of adequate VD levels for sexual health, well-designed randomized controlled trials are essential to delineate causal relationships and potential for therapeutic efficacy.

## 1. Introduction

According to the World Health Organization, erectile dysfunction (ED) is defined as the inability or significant difficulty in achieving or maintaining a penile erection of sufficient rigidity and duration to permit satisfactory sexual performance [1]. The etiology of ED can be psychogenic, organic or mixed. Organic ED may result from neurogenic, hormonal, arterial, or cavernosal dysfunction, and it can also be drug-induced [2]. In 2012, the prevalence of self-reported ED in the past 6 months among adult men in five European countries (France, Germany, Italy, Spain and UK) was 17%, with a clear age-dependent increase observed [3]. In comparison, a 2022 study conducted in Poland reported a markedly higher prevalence of 61.1% among men over 18 years, based on IIEF-5 (International Index of Erectile Function-5) score of 21 point or less, which reflects the presence of any degree of ED. When applying a more stringent threshold of ≤16, indicating moderate to severe ED, the prevalence was 30.1% [4].

There is a strong association between ED and cardiovascular disease (CVD), as ED is increasingly recognized as an early indicator of underlying vascular pathology [5]. Both conditions share common risk factors, including diabetes mellitus, hypertension, and atherosclerosis, which contribute to endothelial dysfunction affecting, among others, the penile vasculature [6]. Accumulating evidence suggests that endothelial function may be influenced by vitamin D (VD) status [5,7]. VD regulates nitric oxide synthesis, thereby promoting vasodilation. It also exerts anti-inflammatory effects by enhancing anti-inflammatory cytokines, reducing pro-inflammatory cytokines, and downregulating the renin-angiotensin system. In addition, impaired VD activity is associated with increased production of reactive oxygen species and diminished antioxidative capacity, both of which may contribute to the development of endothelial dysfunction [8,9].

The relationship between VD and ED may also be modulated by hormonal pathways, as several studies have reported an association between VD deficiency and lower testosterone concentrations [10,11].

In addition, lifestyle factors—such as smoking, physical activity, alcohol consumption, body weight, and sun exposure—may influence both VD and erectile function, and therefore should be considered when interpreting the link between VD and ED [12].

Despite its multifaceted physiological roles, VD deficiency remains a global public health concern. In Europe, standardized data from 2016 indicated that 40.4% of the population had mean annual serum 25(OH)D concentrations below 50 nmol/L, while 13.0% had levels below 30 nmol/L [13]. Given that VD deficiency is associated with CVD risk factors, it has been hypothesized that VD supplementation may help decrease vascular damage and, in turn, reduce the risk of ED [6]. This underscores the importance of investigating the potential association between VD levels and ED.

In recent years, there has been growing interest in the potential connection between VD levels and ED; however, this relationship remains underexplored. This article aims to summarize the existing literature on the relationship between VD levels and ED.

## 2. Materials and Methods

This systematic literature review was performed at the Lithuanian University of Health Sciences, between January and June 2025. This review was registered in the International Prospective Register of Systematic Reviews (PROSPERO, registration code: CRD420251179740). The systematic review was conducted in accordance with Preferred Reporting Items for Systematic Reviews (PRISMA) guidelines (Supplementary Table S1). The Participants-Intervention/Exposure-Comparison-Outcomes (PICO) structure was used to manage the review.

The aim of this review was to present summarized data, including a new clinical trial, on the associations between VD and ED, and to determine the probable critical threshold of VD.

### 2.1. Search Strategy and Selection Criteria

Literature searches were conducted, with a combination of descriptors identified using Medical Subject Headings (MeSH) terms and keywords: ‘vitamin d’, ‘25-hydroxy-Vitamin D’, ‘25 hydroxycholecalciferol’, ‘cholecalciferol’, ‘erectile dysfunction’ (or ‘function’), ‘hypogonadism’, ‘impotence’, ‘PDE5 inhibitors’ in the electronic PubMed and the Cochrane Library databases. The exact search strings are reported in the manuscript Supplementary Material Table S5. The period for the search was from 1 January 2010 to 1 January 2025. Inclusion criteria were: clinical trial, prospective observational and retrospective observational studies, conducted in general male adult population (with the exclusion of special patient populations, including individuals with type 2 diabetes mellitus and chronic kidney disease, which are discussed separately) and articles in English language only. The negative filters applied to the databases were studies available only in full text, as well as narrative or systematic reviews and meta-analyses, which were used solely to support the research background and discussion.

### 2.2. Evaluation of Studies

Two authors separately investigated the quality and acceptability of the articles. Any disagreement between them was resolved by the third author. Agreement between the two authors was assessed using Cohen’s kappa (κ = 0.83). Duplicates were identified and removed using EndNote’s (version: 22.0.0.19000) duplicate detection tools.

### 2.3. Data Extraction and Quality Assessment

Information extrapolated from the selected studies was collected in a Microsoft Excel spreadsheet, by the reviewers. The risk of bias was independently assessed using the Newcastle–Ottawa scale (NOS) (for observational studies). NOS was used to assess the quality of case–control and cohort studies based on its standard nine-point scoring system. An adapted version of NOS was applied to cross-sectional studies.

A meta-analysis was not performed due to substantial heterogeneity across studies, including differences in 25(OH)D assay methods and reference ranges, variations in study populations, and inconsistent definitions and assessments of ED severity.

All *p*-values lower than 0.05 were considered statistically significant. The strength of the correlation was interpreted as weak (r = 0.2–0.3), moderate (r = 0.3–0.5), strong (r = 0.5–0.7), and very strong (r ≥ 0.7).

There was no funding source for this study.

## 3. Results

### 3.1. Search Results

**Figure 1:**
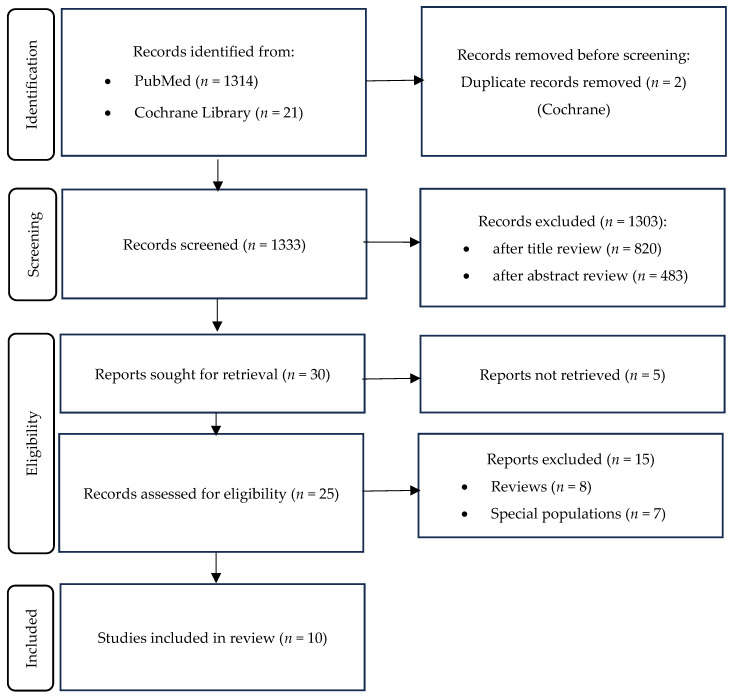
Search strategy flowchart.

A total of 1335 articles were identified from 2 databases: 1314 from PubMed and 21 from Cochrane (Figure 1). After screening, 30 articles were selected for full text review, of which 20 were excluded (reasons listed in Figure 1). Data extraction was performed in 10 studies: 2 RCTs, 1 pilot clinical study, 1 case–control study, 4 cross-sectional studies, and 2 retrospective studies. Together, they encompassed over 13,000 participants. The main findings of the included studies are summarized in Table 1, with emphasis on study populations, assessment methods, and key outcome measures.

### 3.2. Assessment of Vitamin D Serum Concentration, Optimal Value and Cut-Off Value

*Assessment of Vitamin D Status.* Different methodologies were used to evaluate VD levels across the analyzed studies. In the D-Health trial (Romero et al. [14]), baseline 25(OH)D concentrations were estimated, and only post-supplementation VD levels were directly measured. In the other studies included in this review, VD concentrations were determined through direct measurement. The specific analytical techniques utilized are summarized in Table 1.

*Optimal Vitamin D Concentration.* The definition of optimal VD levels (the value selected by the authors) varied slightly across studies, ranging from ≥50 to ≥75 nmol/L. In the randomized controlled trial conducted by Romero et al. [14], the optimal predicted threshold for VD was defined as ≥50 nmol/L. After three years of participation, the median serum VD level in both groups was ≥75 nmol/L. The mean serum 25(OH)D concentration was 76 ± 24.94 nmol/L in the placebo group and 106 ± 26.76 nmol/L in the VD group (*p* < 0.0001). In a cross-sectional study by Zhang et al. [20], the optimal VD concentration was defined as >62.5 nmol/L. Four of the ten studies (Dumbraveanu et al., Wu et al., Barassi et al., and Farag et al. [18,19,21,22]) identified an optimal level exceeding 75 nmol/L. The studies by Horsanali et al., Culha et al., Yang et al. and Ermec at al. [16,17,23,24] did not specify reference values for optimal VD concentration.

*Vitamin D Cutoff Values for Assessing Association with Erectile Dysfunction.* The VD cutoff points (thresholds at which significant associations with ED were identified) varied among the studies, ranging from 37.5 nmol/L to 75 nmol/L (Table 2). In the D-Health trial [14], baseline data indicated that most participants in both groups (79.6%) had predicted 25(OH)D concentrations above 50 nmol/L, with no further stratification of individuals below this threshold into smaller cutoff categories, no statistically significant differences were found.

### 3.3. Assessment of Erectile Function

*Questionnaires used for assessment of Erectile Function.* In most of the included studies [16,17,18,19,20,21,23,24] ED was evaluated using either the SHIM (Sexual Health Inventory for Men), the IIEF-5, or the IIEF-EF. The SHIM and IIEF-5 are abbreviated versions of the IIEF-15 (International Index of Erectile Function), each comprising five questions, whereas the IIEF-EF includes six questions. When interpreting the results of ED questionnaires, some researchers (Dumbraveanu et al., Barassi et al. [18,21]) included a ‘mild to moderate’ and ‘complete’ (considered when IIEF-5 score ≤ 4) categories, while all the others classified ED into three categories: severe (0–7 points), moderate (8–14 points), and mild (15–21 points). In most studies, an IIEF-5 score below 21 was used to identify varying degrees of ED. In one study (Culha et al. [24]), scores ranging from 17 to 25 were classified as mild ED, whereas scores between 0 and 16 were categorized as moderate to severe ED. In the randomized controlled trial, ED was assessed using a single question. Similarly, in the study by Farag et al., ED was evaluated through a single item derived from the Massachusetts Male Aging Study (MMAS).

*Additional diagnostic testing for the etiology of Erectile Dysfunction.* To differentiate the etiology of ED, four of the included studies [19,20,21,24] conducted additional diagnostic evaluations. In the study by Wu et al. [19], nocturnal penile tumescence and rigidity (NPTR) testing was utilized: patients exhibiting at least one effective erectile event during two consecutive nights were diagnosed with psychogenic ED, whereas those without such events were diagnosed with organic ED. Penile blood flow was further assessed using Doppler ultrasonography in the study by Barassi et al. [21]. In the studies conducted by Zhang et al. and Culha et al. [20,24], additional parameters such as carotid intima-media thickness (CIMT) and mean platelet volume (MPV) were measured.

### 3.4. Risk of Bias Assessment

The NOS consists of three categories: selection, comparability, and outcome/exposure. Cross-sectional studies were scored on a 0–10 scale, with scores ≥ 7 classified as high quality, scores of 5–6 as moderate quality, and scores ≤ 4 as low quality. Detailed criteria for risk-of-bias assessment are presented in Supplementary Table S2. The risk of bias in randomized controlled trials is summarized in Supplementary Table S3. A study is classified as having a high risk of bias if one or more domains are rated as high risk.

### 3.5. Main Findings on the Association Between Vitamin D Levels and Erectile Dysfunction

In the majority of reviewed studies, the findings highlighted the importance of VD in ED, especially in men with moderate to severe or complete arteriogenic ED (A-ED). In the study by Wu et al., the prevalence of A-ED was 60.3% in patients with 25(OH)D deficiency compared with 23.7% in those without the deficiency (*p* < 0.001). The summarized results from the analyzed studies are presented in Table 2. In all of the studies, except by Romero et al. [14], patients with severe or complete ED had significantly lower VD levels compared to those with mild ED (detailed results are presented in Table 2). This association was especially evident in studies that included additional methods for evaluating vascular function, such as penile echo-color Doppler, ultrasound or measurement from complete blood count. Penile echo-color Doppler analysis demonstrated that arteriogenic ED was more frequent in individuals with VD deficiency compared with those with serum VD levels > 50 nmol/L (>20 ng/mL) (45% vs. 24%, respectively; *p* < 0.05).

A study by Zhang et al. [20] investigating the associations between serum 25(OH)D levels, CIMT, and IIEF-5 scores reported similar findings. Even after adjusting for potential confounding factors, significant associations remained (r = 0.430). Specifically, serum 25(OH)D levels positively correlated with IIEF-5 scores, indicating better erectile function with higher VD levels, whereas CIMT values were negatively correlated with IIEF-5 scores, suggesting that increased arterial thickness is associated with poorer erectile function.

In a study by Barassi et al. [21] that additionally evaluated peak systolic velocity (PSV), patients with 25(OH)D deficiency exhibited significantly lower IIEF-5 scores compared to those without deficiency. Similarly, PSV values were markedly reduced in the deficiency group, with both differences reaching high statistical significance (*p* < 0.001).

In a study by Culha et al. [24], MPV values were significantly higher in patients with moderate and severe ED compared with those with mild ED, and 25(OH)D levels were significantly lower in the moderate and severe ED groups. A moderate negative correlation (r = −0.23) was observed between 25(OH)D levels and MPV values.

In the studies by Dumbraveanu et al., Wu et al., Zhang et al. and Ermec et al. [17,18,19,20] strong correlations between VD levels and the severity of ED were demonstrated. Other studies revealed a nonsignificant trend indicating lower mean VD levels with increasing ED severity. Across all analyzed studies, median IIEF-5 scores were consistently and significantly lower in patients with reduced VD levels. These findings collectively highlight that VD deficiency is common among men with ED and is particularly prevalent in cases with an arteriogenic etiology.

Noteworthy results were observed in the study with one of the largest sample sizes—the NHANES cohort [22]. In the NHANES study, men with ED had significantly lower serum 25(OH)D levels compared to those without ED. After adjusting for lifestyle factors, comorbid conditions, and medication use, VD deficiency was associated with a higher prevalence of ED compared to men with serum 25(OH)D levels > 75 nmol/L (≥30 ng/mL). In fully adjusted models, 25(OH)D levels below 50 nmol/L (20 ng/mL) were linked to increased odds of prevalent ED, while levels above 87.5 nmol/L (35 ng/mL) corresponded with a reduced prevalence. This cross-sectional analysis indicates that VD deficiency is independently associated with increased ED prevalence, regardless of lifestyle, atherosclerotic cardiovascular disease (ASCVD) risk factors, or sex hormone levels [22].

Meanwhile, interventional studies showed mixed results regarding the effect of VD supplementation for ED. Ermec et al. found that VD replacement combined with daily tadalafil (5 mg) improved IIEF-EF scores from 10.73 ± 6.12 at baseline to 24.18 ± 4.87 post-treatment (*p* = 0.001). In another comparative study, the intervention group receiving VD plus sildenafil had mean serum 25(OH)D levels of 45.37 ± 5.48 ng/mL, compared to 20.13 ± 4.06 ng/mL in the control (sildenafil alone) group (*p* < 0.05). The number of patients with significantly effective treatment was 31 (38.75%) in the experimental group versus 12 (15.58%) in the control group (*p* = 0.03)

Conversely, the largest randomized controlled trial to date investigating VD supplementation found no significant difference in the prevalence of ED between the VD and placebo groups, with rates of 58.8% and 59.0%, respectively, following supplementation [14].

In studies evaluating metabolic parameters, including lipid profiles [18,19,20,21,23,25] patients with organic ED were categorized according to their 25(OH)D status. In most studies, no statistically significant differences were observed between groups in clinical or metabolic variables such as age, BMI, testosterone, estradiol, LH, FSH, or lipid profiles. However, the study by Canguven et al. [26] reported distinct findings: ergocalciferol supplementation produced a significant increase in serum total testosterone (from 12.46  ±  3.30 to 15.99  ±  1.84 nmol/L, *p* < 0.001) and a significant reduction in BMI at 12 months (from 33.91  ±  6.67 to 33.14  ±  6.35 kg/m^2^, *p* = 0.001). This study also found significant decreases in low-density lipoprotein cholesterol (from 2.91  ±  0.86 to 2.68  ±  0.42 mmol/L, *p* = 0.001) and triglycerides (from 1.61  ±  0.51 to 1.44  ±  0.46 mmol/L, *p* = 0.035), whereas the change in total cholesterol was not statistically significant.

The main results of the studies included are summarized in Table 2.

**Table 2:** Main results of the included studies.

No.	Primary Outcome	Intervention	BMI (kg/m^2^)	Testosterone (nmol/L)	25(OH)D Cut-Off Value (nmol/L)	Findings	Correlation	Proposed Vit. D Level (nmol/L) for Decreased ED Risk
1. [14]	ED prevalence after 3 years of suppl.	VD (60,000 IU/month) vs. placebo	Placebo: <25—26.5%; 25–30—49.5%; ≥30—24.0% VD: <25—26.0%; 25–30—49.4%; ≥30—24.6%	–	<50 predicted baseline; >75 post suppl.	No difference in ED prevalence. VD suppl. ineffective for ED in older men	-	-
2. [16]	Efficacy: VD + sildenafil (S) vs. S	VD 400 IU/d + S 100 mg vs. S 100 mg	VD + S: 27.33 ± 1.66. S only: 27.12 ± 1.63	VD + S: 11.42 ± 2.76. S only: 11.63 ± 2.58	-	VD improves S response (*p* < 0.05).	-	-
3. [17]	VD suppl. in Tadalafil (T) non-responders	VD 150,000 IU + T 5 mg/d	Mean: 27.55 ± 3.95	Mean: 8.49 ± 3.43	-	IIEF-EF ↑, 25(OH)D ↑. VD suppl. reduced ED in T non-responders	IIEF-EF scores and 25(OH)D post suppl.: strong correlation (r = 0.661, *p* < 0.001)	-
4. [18]	VD level and ED severity	–	Mean: 26.84 ± 2.96; ED: 26.90 ± 2.97; no ED: 26.67 ± 3.03	ED: 16.1 ± 6.01; No ED: 16.9 ± 7.07	>75	(25OH)D ↓ in severe ED. Higher 25(OH)D = less severe ED	(25OH)D and ED severity: strong correlation (ρ = 0.752, *p* < 0.001)	-
5. [19]	25(OH)D and ED (arteriogenic (A-ED))	–	Mean: 24.07 ± 3.73; organic ED: 24.35 ± 3.86; psychogenic ED: 23.51 ± 3.44	Mean: 15.12 ± 5.19; Organic ED: 14.13 ± 4.14 Psychogenic ED: 15.60 ± 5.59	37.625	Lowest 25(OH)D in A-ED	IIEF-5 scores and 25(OH)D: strong correlation (r = 0.653, *p* < 0.001)	-
6. [20]	25(OH)D, CIMT, and ED severity	–	Mild ED: 23.62 ± 3.15; moderate: 23.19 ± 2.27; severe: 23.81 ± 3.14; controls: 23.46 ± 2.88	Abnormal level—exclusion	<50	25(OH)D ↓ in moderate/severe ED. 25(OH)D linked to ED severity and CIMT	IIEF-5 scores and 25(OH)D: strong correlation (r = 0.430, *p* < 0.05)	-
7. [21]	25(OH)D in ED subtypes	–	A-ED: 23.8; BL-ED: 24.3; NA-ED: 23.2	A-ED: 410 ng/mL; BL-ED: 470 ng/mL; NA-ED: 454 ng/mL.	>50	Higher VD deficiency in A-ED	-	>75
8. [22]	25(OH)D levels and ED prevalence	–	ED: 29.0 ± 0.33; no ED: 27.83 ± 0.13	–	≥50	Lower 25(OH)D in ED group. ED prevalence ↑ in 25(OH)D deficiency	25(OH)D deficiency—higher ED prevalence (PR = 1.30, 95% CI 1.08–1.57)	>87.5
9. [23]	ED severity and 25(OH)D	–	Mild ED: 26.70 ± 4.05; moderate: 27.58 ± 3.59; severe: 28.66 ± 3.58.	Mild ED: 18.0 ± 4.7; moderate: 16.2 ± 6.1, severe: 14.6 ± 6.9	68.3	25(OH)D levels were associated with the severity of ED: lower 25(OH)D in severe ED.	25(OH)D and ED: positive correlation (r = 0.193, *p* = 0.028)	>68.3
10. [24]	MPV, 25(OH)D and ED severity	–	Mean: 27.59 ± 3.91	testosterone deficiency—exclusion	>37.5	Lower 25(OH)D in severe ED vs. mild. 25(OH)D predicts ED severity	IIEF-5 scores and 25(OH)D: positive correlation (r = 0.22, *p* = 0.03)	-

VD—vitamin D; ED—erectile dysfunction; 25(OH)D—25-hydroxy-vitamin-D; CIMT—carotid artery intima-media thickness; MPV—mean platelet volume; BMI—body mass index; A-ED—arteriogenic ED; BL-ED—borderline ED; NA-ED—non-arteriogenic ED; IIEF-EF—International Index of Erectile Function-Erectile Function domain; IIEF-5—International Index of Erectile Function-5; ↑: increased; ↓: decreased.

### 3.6. GRADE Summary of Findings

Due to the heterogeneity of the included study designs (cross-sectional, case–control, retrospective, and randomized clinical trials), it is essential to provide a clear certainty-of-evidence assessment. Therefore, Supplementary Table S4 presents a certainty-of-evidence evaluation following the GRADE framework. A GRADE-based assessment has been incorporated for each main outcome, including ED prevalence, ED severity, vascular ED, and supplementation effects (Supplementary Table S4).

### 3.7. Special Populations

The associations between ED and VD have also been analyzed in specific populations. Considering our selected inclusion and exclusion criteria, these studies were not included in detailed analysis; however, the main aspects are discussed below.

*Type 2 Diabetes.* In the study by Basat et al. [25], 98 patients with type 2 diabetes mellitus were included and divided into three groups according to their IIEF-5 scores. Serum 25(OH)D levels were significantly lower in patients with severe ED (*p* = 0.020). Moreover, a moderate positive correlation was observed between the IIEF-5 score and 25(OH)D level (r = 0.21, *p* = 0.038) [25]. Similar findings were reported in the study by Caretta et al. [27]: patients with type 2 diabetes mellitus and lower 25(OH)D levels (<25 nmol/L) had significantly lower IIEF-5 scores (*p* < 0.005), testosterone concentrations (*p* < 0.05), and cavernous PSV (*p* < 0.05) compared with patients whose 25(OH)D levels were >50 nmol/L. Moreover, 25(OH)D levels were directly correlated with IIEF-5 scores (r = 0.39; *p* = 0.0001) [27]. In the study by Canguven et al. [26], ergocalciferol supplementation led to a significant improvement in erectile function scores, increasing from 13.88 ± 3.96 at baseline to 20.25 ± 3.24 after twelve months (*p* < 0.001).

The results were further confirmed by a meta-analysis published in 2022, which included four studies. The analysis showed that VD levels in patients with both diabetes and ED were significantly lower (12.5 nmol/mL) than in patients with diabetes alone (*p* = 0.002) [28].

In the study by Raharinavalona et al. [29], analyzing 155 men aged >55 years with type 2 diabetes to identify the best biological marker for predicting ED, findings differed from those described above: VD levels did not differ significantly between men with and without ED (82.35 ± 33.33 vs. 97.13 ± 34.33 ng/mL, *p* = 0.14). However, it should be noted that in this study, ED was not classified according to severity, which may have influenced the results [29].

*Chronic kidney disease.* The prevalence of ED is extremely high—around 80%—among patients with chronic kidney disease [30]. As a result, the relationship between 25(OH)D levels and ED has been explored in renal transplant recipients; however, studies have reported no significant differences in 25(OH)D concentrations between those with and without ED [31].

*Older men.* A cross-sectional study by Kim et al. in elderly patients (mean age 60.1 years) with untreated lower urinary tract symptoms found a prevalence of 48.7% moderate to severe ED and 54.3% VD deficiency. Age over 60 years and moderate to severe LUTS were identified as independent risk factors for ED [32].

## 4. Discussion

Numerous studies have investigated the association between VD status and ED, but the current body of evidence remains fragmented, with inconsistent findings and a limited number of randomized controlled trials. These inconsistencies contribute to ongoing debate and are echoed in the present review.

The physiological basis for the relationship between VD and ED lies in VD’s regulatory role in endothelial function, mainly through enhancing nitric oxide synthesis, reducing pro-inflammatory cytokines, and modulating oxidative stress pathways [6,7]. These mechanisms facilitate penile vasodilation critical for erection. Supporting this, studies employing penile Doppler ultrasound and carotid intima-media thickness (CIMT) measurements found VD deficiency correlated with impaired penile blood flow and increased arterial stiffness, both linked to ED severity [20,21].

Although VD may exert a direct effect on ED via endothelial pathways, an indirect pathway mediated by testosterone is also plausible as several studies have reported associations between VD and testosterone levels [33,34]. In a combined cross-sectional and longitudinal study, Tirabassi et al. found that VD supplementation was accompanied by increase in total and free testosterone levels together with improvements in erectile function, although its observational design limits causal interference [35]. Notably, several studies included in our systematic review provide further support for hormone-mediated pathway: Yang et al. reported significantly increased testosterone levels and improved ED in a group receiving both VD and sildenafil [16]. Additionally, Canguven et al. showed that VD treatment improved metabolic parameters, such as glycated hemoglobin, and increased testosterone concentrations [26]. Taken together, these findings suggest that VD may influence erectile function through both direct mechanisms and hormone-mediated pathways.

Two systematic reviews published in 2019 and 2020 [36,37] concluded that VD deficiency may be linked to more severe forms of ED. While some overlap exists between the included studies from these earlier reviews and those analyzed in the present review, several key differences are noteworthy. Most of the studies selected for this review excluded participants with severe chronic diseases, and importantly, free interventional trials were included, whereas previous systematic reviews focused primarily on observational research. One of these three trials is the largest randomized clinical trial of VD supplementation for ED to date.

The majority of the studies we reviewed have highlighted the role of VD in supporting erectile function, particularly in cases of moderate to severe arteriogenic ED (A-ED); however, it is important to note that most of these findings are derived from observational studies providing moderate-quality evidence. Randomized controlled trials (RCTs) examining VD supplementation efficacy on ED have yielded mixed outcomes. The largest RCT to date, the D-Health trial, reported no significant difference in ED prevalence between the VD and placebo groups, with prevalence at 58.8% and 59.0%, respectively (prevalence ratio 1.00, 95% CI 0.97–1.03). This outcome indicates either the absence of a causal relationship or a more complex interaction between VD status and ED that was not adequately captured by the study’s design.

While the D-Health trial provides valuable large-scale evidence, its methodology may have obscured potential effects. All participants generally maintained sufficient VD levels throughout the trial, with the placebo group’s mean serum 25(OH)D at about 76 nmol/L—within the ‘optimal range’ as noted by the observational studies. This restricted the ability to test for benefits in truly deficient populations who may be more responsive to supplementation. Notably, participants in the D-Health trial were stratified into groups with VD levels below and above 50 nmol/L based on predicted values generated by algorithms that accounted for season, geographic location, and participant characteristics, rather than direct serum measurement at baseline. This methodology may introduce estimation errors, as algorithm-based prediction is less precise compared to direct biochemical measurement. Such estimation can affect the reliability of baseline classification and potentially influence study outcomes. Furthermore, the trial did not specifically target men with VD deficiency and ED, potentially diluting treatment effects by including a broad participant pool. Moreover, a high-dose monthly VD regimen (60,000 IU/month) was used, which might not reflect physiological VD metabolism as closely as daily dosing. Finally, erectile function in this study was assessed at a single follow-up time point using one self-report question, rather than a validated questionnaire or severity classification, which could reduce assessment accuracy. Similar limitations were present in studies such as the Massachusetts Male Aging Study, where ED was evaluated using a single direct question, potentially impacting validity and reliability. Results in a study by Kim et al. in elderly men highlight that the methodology used for ED assessment—especially regarding questionnaire validity and severity classification—can notably impact observed relationships, and that more granular approaches are preferable in future research [32].

In contrast, smaller interventional studies have shown promising improvements in erectile function when VD supplementation was combined with PDE5 inhibitors in VD-deficient men [16,17]. These factors contribute to the interpretation challenges and underscore the need for future trials focused on deficient individuals and with more precise dosing strategies.

The stronger relationship between VD deficiency and ED in certain populations, such as men with type 2 diabetes mellitus [25,27], underlines the clinical relevance of VD in vascular-related ED. Meta-analyses pooling these data confirm that diabetic men with ED have significantly lower VD levels than those without ED [28]. Given the high prevalence of both VD deficiency and endothelial dysfunction in metabolic disorders, VD supplementation might offer a beneficial adjunctive approach, although targeted clinical trials in this group are necessary.

When interpreting the relationship between VD and ED, potential lifestyle-related confounders should be considered. Physical activity, dietary habits, smoking, and alcohol consumption may influence both VD status and ED, yet most included studies did not adequately assess these behaviors. Obesity is another confounder, as it affects endothelial function and is consistently associated with lower VD levels [38]. Among the included studies, only three specifically examined the association between VD status and BMI. Dumbraveanu et al. reported a significant negative correlation between VD and BMI (*p* = −0.517, *p* < 0.001), whereas Barassi et al. and Wu et al. did not find significant difference in BMI between VD deficient and non-deficient participants. Hypogonadism, as mentioned before characterized by reduced testosterone levels, has also been linked to diminished VD status and more severe ED [34,39], only a subset of studies accounted for this factor. Additionally, seasonal variation and sun exposure substantially affects VD levels and should be considered when evaluating the observed associations [40]. It should also be noted that in randomized clinical trials, as well as in routine clinical practice, priority should be given to pharmaceutical vitamin D preparations that ensure standardized dosing, concentration and allow for more accurate and safer vitamin D correction [41]. Therefore, these confounders should be carefully considered when interpreting the association between VD and ED.

Methodological heterogeneity across studies represents an important limitation, substantially restricting their direct comparability. Specifically, vitamin D measurement employed diverse methodologies (LC–MS/MS, ELISA, CLIA, CMIA, RIA), and vitamin D deficiency thresholds varied substantially (37.5–75 nmol/L). Similarly, erectile dysfunction assessment relied on both validated instruments (IIEF-5, IIEF-EF, SHIM) and single-item questions. Even among studies utilizing identical assessment tools, disease severity classifications were inconsistently applied, introducing additional variability in outcome measurement. Notably, the heterogeneity in ED assessment instruments across included studies limits our ability to distinguish between ED severity and etiology. Specifically, addressing the relationship between vitamin D levels and arteriogenic erectile dysfunction would require more granular analyses from rigorously designed studies employing objective vascular assessment. Only one included study (Barassi et al.) assessed penile blood flow using duplex Doppler ultrasonography, which constrains our capacity to differentiate arteriogenic from non-arteriogenic etiologies and their respective associations with vitamin D status. Although vitamin D deficiency was independently associated with erectile dysfunction irrespective of cardiovascular risk factors, penile arterial insufficiency and systemic cardiovascular disease represent related but distinct pathophysiological processes. Our findings do not preclude a potentially stronger or more specific relationship between vitamin D status and arteriogenic erectile dysfunction, underscoring the need for targeted investigation employing direct vascular assessment in this subpopulation. Furthermore, the predominance of cross-sectional designs fundamentally limits causal inference. Collectively, this heterogeneity constrains the direct comparison of findings and precludes the formulation of robust clinical recommendations.

In summary, among the studies reviewed, calculated VD cut-off values used to assess differences in ED varied somewhat, generally ranging from 37.6 to 75 nmol/L, as presented in Table 2. Notably, a lower prevalence of ED was most consistently associated with serum VD concentrations above 75–87.5 nmol/L (equivalent to 30–35 ng/mL). These findings should be interpreted cautiously, as the evidence for supplementation remains inconclusive. Pooled estimates or definitive thresholds cannot be derived, since these ranges are based on low-certainty evidence from a limited number of studies, most of which are observational or small interventional studies. Well-designed randomized controlled trials are needed to provide more reliable estimates and to clarify whether the observed associations reflect true causal effects. Notably, there still remains a lack of evidence on whether VD’s impact on ED differs in diabetic vs. non-diabetic patients, across different age groups, or according to severity of VD deficiency; these subpopulations might warrant focused attention in future studies.

## 5. Conclusions

This systematic review highlights an association between vitamin D status and erectile dysfunction, particularly in men with moderate-to-severe or arteriogenic ED. Lower vitamin D levels were independently correlated with higher ED prevalence, irrespective of confounding factors such as lifestyle, cardiovascular risks, and hormonal status.

The evidence from several observational studies suggests that maintaining serum vitamin D concentrations above 75–87.5 nmol/L (30–35 ng/mL) may be related to improved endothelial function and sexual health outcomes. A clear causal relationship cannot be established, given the low certainty of the evidence from observational studies. Despite the observational data, current randomized controlled trials provide inconclusive evidence regarding the therapeutic benefit of vitamin D supplementation in improving erectile function.

This underscores the necessity for well-designed, randomized, placebo-controlled trials focusing on individuals with severe vitamin D deficiency and arteriogenic ED to establish causality and clarify treatment efficacy.

Overall, optimizing vitamin D status may represent a promising avenue for improving erectile dysfunction, but further rigorous investigation with causality clarification is essential to translate these findings into clinical practice recommendations.

## Figures and Tables

**Table 1:** Basic characteristics of the included studies.

No.	Authors, Year	Country	Study Design	Study Population	Age Range, Mean Age	Sample Size (*n*)	Method and Scoring System for ED Evaluation	Method and Optimal Values (nmol/L) for VD
1.	Romero et al. (2024) [14]	Australia	Randomized placebo-controlled	General male population.	60–84	8920	Self-reported ED	Predicted at baseline (<50; ≥50); after supplementation LC–MS/MS [15]
2.	Yang et al. (2023) [16]	China	Prospective randomized controlled open trial	Men with ED ≥ 1 year, 25(OH)D < 75 nmol/L.	41–58, 49.25 ± 5.29	157	IIEF-5	-
3.	Ermec et. al. (2022) [17]	Turkey	Pilot clinical study	Men with ED, Tadalafil 5 mg non-responders, VD < 50 nmol/L.	28–70, 42.15 ± 9.04	84	IIEF-EF: ≤23	CMIA
4.	Dumbraveanu et al. (2020) [18]	Moldova	Case–control	Men with sexual/reproductive complaints.	22–67, 42.96 ± 11.83	84	IIEF-5: <22	CLIA. <25—deficiency; 25–75—insufficiency, 75–250—optimal.
5.	Wu et al. (2022) [19]	China	Cross-sectional	Men with ED > 6 months.	18–60, 37.97 ± 8.83	150	IIEF-5: <21; NPTR; ECD	CLIA. <50—deficiency, 50–75—insufficiency, >75—optimal.
6.	Zhang et al. (2022) [20]	China	Cross-sectional	Physical exam participants.	30–60, 45.41 ± 7.44	163	IIEF-5: <22	ELISA. <50—deficiency; 50–62.5—insufficiency, >62.5—optimal.
7.	Barasi et al. (2014) [21]	Italy	Cross-sectional	Men with ED.	30–60, 47	143	IIEF-5: <21; ECD	RIA. <50—deficiency; 50–75—insufficiency, >75—optimal.
8.	Farag et al. (2016) [22]	USA	Cross-sectional	General male population.	20–85	3390	ED: self-reported	RIA. <50—deficiency, 50–75—insufficiency, ≥75—optimal.
9.	Horsanali et al. (2020) [23]	Turkey	Retrospective	Men with ED.	18–80, 49.28 ± 13.62	130	IIEF-5: <25	CLIA
10.	Culha et al. (2020) [24]	Turkey	Retrospective	Andrology clinic patients.	18–65, 41.07 ± 8.56	90	IIEF-EF: <25	CMIA

The majority of studies excluded participants with cardiovascular, renal, systemic inflammatory, metabolic, or oncologic diseases, as well as individuals with diabetes or those using VD supplements. ED—erectile dysfunction; 25(OH)D—25-hydroxyvitamin D; VD—vitamin D; IIEF-5—International Index of Erectile Function-5; IIEF-EF—International Index of Erectile Function-Erectile Function domain; NPTR—Nocturnal Penile Tumescence and Rigidity; ECD—Echo Color Doppler; LC–MS/MS—Liquid chromatography tandem mass spectrometry; CMIA—Chemiluminescence microparticle immunoassay; CLIA—Chemiluminescence immunoassay; ELISA—Enzyme-Linked Immunosorbent Assay; RIA—Radioimmunoassay.

## Data Availability

No new data were created or analyzed in this study.

## Supplementary Materials

The following supporting information can be downloaded at: https://www.mdpi.com/article/10.3390/jcm14248630/s1, Table S1: PRISMA 2020 checklist; Table S2: Evaluation of risk of bias using the Newcastle Ottawa Scale; Table S3: Cochrane Risk of Bias 2 for randomized clinical trials; Table S4: Summary-of-findings; Table S5: Research strings.

## Abbreviations

The following abbreviations are used in this manuscript:

A-ED	Arteriogenic ED
BL-ED	Borderline ED
BMI	Body mass index
CMIA	Chemiluminescence microparticle immunoassay
CVD	Cardiovascular disease
ECD	Echo-color-Doppler.
ED	Erectile dysfunction
FSH	Follicle-stimulating hormone
IIEF	International Index of Erectile Function
LC–MS/MS	Liquid chromatography tandem mass spectrometry
LH	Luteinizing hormone
LUTS	Lowe urinary tract symptoms
NPTR	Nocturnal penile tumescence and rigidity
MMAS	Massachusetts Male Aging Study
MeSH	Medical Subjects Headings
NA-ED	non-arteriogenic ED
SHIM	Sexual Health Inventory for Men
UK	United Kingdom
VD	vitamin D
25(OH)D	25-hydroxy-vitamin-D
