# Supplementary material for: The Association Between Vitamin D Levels and Erectile Dysfunction in Men: A Systematic Review
Source: J Clin Med. 2025 Dec 5;14(24):8630. doi: 10.3390/jcm14248630 (PMC12733428; doi:10.3390/jcm14248630)
Supplement: Supplementary file 1 [file jcm-14-08630-s001.zip › jcm-3991193-supplementary.pdf]

Supplementary Table S1

Table S1. PRISMA 2020 checklist

Section and Topic	Item #	Checklist item	Location where item is reported
**TITLE**			
Title	1	Identify the report as a systematic review.	Page 1, line 3
**ABSTRACT**			
Abstract	2	See the PRISMA 2020 for Abstracts checklist.	Page 1, line 23
**INTRODUCTION**			
Rationale	3	Describe the rationale for the review in the context of existing knowledge.	Page 2, line 63-89
Objectives	4	Provide an explicit statement of the objective(s) or question(s) the review addresses.	Page 3, line 98-100
**METHODS**			
Eligibility criteria	5	Specify the inclusion and exclusion criteria for the review and how studies were grouped for the syntheses.	Page 3, line 107-114
Information sources	6	Specify all databases, registers, websites, organisations, reference lists and other sources searched or consulted to identify studies. Specify the date when each source was last searched or consulted.	Page 3, line 102-114
Search strategy	7	Present the full search strategies for all databases, registers and websites, including any filters and limits used.	Page 3, line 102-114
Selection process	8	Specify the methods used to decide whether a study met the inclusion criteria of the review, including how many reviewers screened each record and each report retrieved, whether they worked independently, and if applicable, details of automation tools used in the process.	Page 3, line 115-119
Data collection process	9	Specify the methods used to collect data from reports, including how many reviewers collected data from each report, whether they worked independently, any processes for obtaining or confirming data from study investigators, and if applicable, details of automation tools used in the process.	Page 3, line 115-122
Data items	10a	List and define all outcomes for which data were sought. Specify whether all results that were compatible with each outcome domain in each study were sought (e.g. for all measures, time points, analyses), and if not, the methods used to decide which results to collect.	Page 6-7 (Table 1), page 10-11 (Table 2)
	10b	List and define all other variables for which data were sought (e.g. participant and intervention characteristics, funding sources). Describe any assumptions made about any missing or unclear information.	Page 6-7 (Table 1), page 10-11 (Table 2)
Study risk of bias assessment	11	Specify the methods used to assess risk of bias in the included studies, including details of the tool(s) used, how many reviewers assessed each study and whether they worked independently, and if applicable, details of automation tools used in the process.	Page 8, line 220-226
Effect measures	12	Specify for each outcome the effect measure(s) (e.g. risk ratio, mean difference) used in the synthesis or presentation of results.	Page 4, line 129-131
Synthesis methods	13a	Describe the processes used to decide which studies were eligible for each synthesis (e.g. tabulating the study intervention characteristics and comparing against the planned groups for each synthesis (item #5)).	-
	13b	Describe any methods required to prepare the data for presentation or synthesis, such as handling of missing summary statistics, or data conversions.	-
	13c	Describe any methods used to tabulate or visually display results of individual studies and syntheses.	-
	13d	Describe any methods used to synthesize results and provide a rationale for the choice(s). If meta-analysis was performed, describe the model(s), method(s) to identify the presence and extent of statistical heterogeneity, and software package(s) used.	-
	13e	Describe any methods used to explore possible causes of heterogeneity among study results (e.g. subgroup analysis, meta-regression).	-
	13f	Describe any sensitivity analyses conducted to assess robustness of the synthesized results.	-

Section and Topic	Item #	Checklist item	Location where item is reported
Reporting bias assessment	14	Describe any methods used to assess risk of bias due to missing results in a synthesis (arising from reporting biases).	-
Certainty assessment	15	Describe any methods used to assess certainty (or confidence) in the body of evidence for an outcome.	Page 11, line 298-304
**RESULTS**			
Study selection	16a	Describe the results of the search and selection process, from the number of records identified in the search to the number of studies included in the review, ideally using a flow diagram.	Page 4-5, 135-142 (Fig. 1)
	16b	Cite studies that might appear to meet the inclusion criteria, but which were excluded, and explain why they were excluded.	Page 12, line 305-339
Study characteristics	17	Cite each included study and present its characteristics.	Page 6-7 (Table 1)
Risk of bias in studies	18	Present assessments of risk of bias for each included study.	Page 8, line 220-226 (Suppl. Table 2, 3)
Results of individual studies	19	For all outcomes, present, for each study: (a) summary statistics for each group (where appropriate) and (b) an effect estimate and its precision (e.g. confidence/credible interval), ideally using structured tables or plots.	Page 10-11 (Table 2)
Results of syntheses	20a	For each synthesis, briefly summarise the characteristics and risk of bias among contributing studies.	-
	20b	Present results of all statistical syntheses conducted. If meta-analysis was done, present for each the summary estimate and its precision (e.g. confidence/credible interval) and measures of statistical heterogeneity. If comparing groups, describe the direction of the effect.	-
	20c	Present results of all investigations of possible causes of heterogeneity among study results.	-
	20d	Present results of all sensitivity analyses conducted to assess the robustness of the synthesized results.	-
Reporting biases	21	Present assessments of risk of bias due to missing results (arising from reporting biases) for each synthesis assessed.	-
Certainty of evidence	22	Present assessments of certainty (or confidence) in the body of evidence for each outcome assessed.	Page 11, line 298-304 (Supll. Table 4)
**DISCUSSION**			
Discussion	23a	Provide a general interpretation of the results in the context of other evidence.	Page 12-14, line 352-372, 411-417
	23b	Discuss any limitations of the evidence included in the review.	Page 14, line 418-444
	23c	Discuss any limitations of the review processes used.	-
	23d	Discuss implications of the results for practice, policy, and future research.	Page 15, line 463-471, 478-490
**OTHER INFORMATION**			
Registration and protocol	24a	Provide registration information for the review, including register name and registration number, or state that the review was not registered.	Page 3, line 92-94
	24b	Indicate where the review protocol can be accessed, or state that a protocol was not prepared.	-
	24c	Describe and explain any amendments to information provided at registration or in the protocol.	-
Support	25	Describe sources of financial or non-financial support for the review, and the role of the funders or sponsors in the review.	Page 4, line 132
Competing interests	26	Declare any competing interests of review authors.	Page 15, line 495
Availability of data, code and other materials	27	Report which of the following are publicly available and where they can be found: template data collection forms; data extracted from included studies; data used for all analyses; analytic code; any other materials used in the review.	-

Supplementary Tables S2–S4

Table S2. Evaluation of risk of bias using the Newcastle Ottawa Scale.

Newcastle - Ottawa quality assessment scale for case control studies										
No.	Reference	Selection (max 4)				Comparability (max 2)	Exposure (max 3)			Total score
		Definition of cases	Representativeness of cases	Selection of controls	Definition of controls		Assessment of exposure	Same methods of ascertainment for cases and controls	Non response rate	
4.	Dum-braveanu et al. (2020)	1	1	0	1	1	1	1	0	6/9

Adapted Newcastle - Ottawa quality assessment scale for cross-sectional studies									
No.	Reference	Selection (max 6)				Comparability (max 1)	Outcome (max 3)		Total score
		Representativeness of the sample	Sample size	Non-respondents	Ascertainment of exposure	Based on design and analysis	Based on design and analysis	Statistical test	
5.	Wu et al. (2022)	1	1	0	2	0	2	1	7/10
6.	Zhang et al. (2022)	1	1	0	2	1	1	1	7/10
7.	Barasi et al. (2014)	1	0	0	2	0	2	1	6/10
8.	Farag et al. (2016)	1	2	0	1	1	1	1	7/10
9.	Horsanali et al. (2020)	1	0	0	2	1	1	1	6/10
10.	Culha et al. (2020)	1	0	0	2	0	1	1	5/10

Table S3. Cochrane Risk of Bias 2 for randomized clinical trials.

No.	Reference	D1	D2	D3	D4	D5
1.	Romero et al. (2024)	+	+	+	+	+
2.	Yang et al. (2023)	X	–	+	–	–

Domains: D1 – bias arising from the randomization process; D2 – bias due to deviations from intended intervention; D3 – bias due to missing outcome data; D4 – bias in measurement of the outcome; D5 – bias in selection of the reported results. X high risk, – some concerns, + low risk.

Table S4. Summary-of-findings.

Outcome	Number of studies and study design	Participants (n)	Findings summary	Certainty of evidence (GRADE)	Reasons for rating
ED prevalence	2: RCT, cross-sectional	12310	No effect of VD vs placebo on ED prevalence (APR 1.00); observational data show higher ED prevalence in VD deficiency.	Moderate	Downgraded 1 level for inconsistency/indirectness (large RCT negative but observational data show association).
ED severity	5: cross-sectional, case-control	617	Higher 25(OH)D consistently associated with higher IIEF/IIEF-5 scores.	Low	Remain downgraded for risk of bias, residual confounding, and some imprecision.
Arteriogenic ED	2: cross-sectional	293	Lower 25(OH)D in observed in arteriogenic ED vs non-arteriogenic ED.	Low	Kept low for risk of bias, confounding by cardiovascular risk factors, and limited number of studies.
Supplementation effects	3: RCTs, pilot clinical study	9161	Mixed results. Large RCT: no effect; small trials: improved IIEF and testosterone with VD (often combined with PDE5i).	Low	Overall downgrade for inconsistency, risk of bias (open, non-blinded designs; co-interventions with PDE5i), and imprecision (small trials).

Supplementary Table S5

Table S5. Research strings

Search strings	
Pubmed search strings	(("Vitamin D"[Mesh] OR "25-hydroxy-Vitamin D" OR "25 hydroxycholecalciferol" OR "cholecalciferol") AND ("Erectile Dysfunction"[Mesh] OR "erectile dysfunction" OR "erectile function" OR "hypogonadism" OR "impotence" OR "PDE5 inhibitors"))
Cochrane research strings	("Vitamin D" OR "25-hydroxy-Vitamin D" OR "25 hydroxycholecalciferol" OR "cholecalciferol") AND ("Erectile Dysfunction" OR "erectile dysfunction" OR "erectile function" OR "hypogonadism" OR "impotence" OR "PDE5 inhibitors")

All searches were conducted in the Title, Abstract, and Keyword fields of the respective databases.
